# *Sechium edule* var. *nigrum spinosum* (Chayote) Increases the mRNA Expression of Genes Encoding Sirtuins in Older Adults with Type 2 Diabetes Mellitus

**DOI:** 10.3390/molecules31071182

**Published:** 2026-04-02

**Authors:** Graciela Gavia-García, David Hernández-Álvarez, Taide Laurita Arista-Ugalde, Itzen Aguiñiga-Sánchez, Edelmiro Santiago-Osorio, Jorge Cadena-Iñiguez, Juana Rosado-Pérez, Víctor Manuel Mendoza-Núñez

**Affiliations:** 1 Research Unit on Gerontology, FES Zaragoza, National Autonomous University of Mexico, Mexico City 09230, Mexico; graciela_gavia_garcia@comunidad.unam.mx (G.G.-G.); hereda2912@gmail.com (D.H.-Á.); tdlarista@comunidad.unam.mx (T.L.A.-U.); 2 Hematopoiesis and Leukemia Laboratory, Research Unit on Cell Differentiation and Cancer, FES Zaragoza, National Autonomous University of Mexico, Mexico City 09230, Mexico; itzen.aguiniga@zaragoza.unam.mx (I.A.-S.); edelmiro@unam.mx (E.S.-O.); 3 Postgraduate College, Campus San Luis Potosi, Salinas de Hidalgo, San Luis Potosi 78600, Mexico; jocadena@colpos.mx

**Keywords:** *Sechium edule*, type 2 diabetes mellitus, aging, sirtuins, mRNA

## Abstract

Consumption of *Sechium edule* var. *nigrum spinosum* has antioxidant and hypoglycemic effects. Regarding the former, certain signaling pathways that influence these effects have already been proposed; however, the underlying molecular mechanisms of the hypoglycemic effects remain unknown. It has been recognized that the sirtuin-mediated signaling cascade responds to various stressors, such as oxidative stress, and regulates glucose metabolism. Therefore, it would be of great interest to determine whether there is a link between these two properties and whether it is mediated by sirtuins. Hence, the present study aimed to evaluate the effect of *Sechium edule* on the gene expression of the sirtuin family (SIRT1-SIRT6) in individuals with type 2 diabetes mellitus (T2DM). A quasi-experimental study was conducted with a convenience sample of 26 older adults diagnosed with T2DM, divided into a (i) placebo group (PG; *n* = 14) and (ii) experimental group (EG; *n* = 12). Clinical, biochemical, and anthropometric measurements were performed, and total oxidant/antioxidant capacity (TOS/TAS) and mRNA expression of genes encoding sirtuins were determined. All parameters were measured at baseline, three months, and six months after the intervention. In the EG, the expression levels of SIRT1, SIRT3, SIRT5, and SIRT6 increased by 52%, 69%, 62%, and 69%, respectively, six months after treatment. A 50% decrease in TOS and a 44% increase in TAS were also observed. Our findings suggest that the bioactive components of *Sechium edule* enhance sirtuin expression and exhibit antioxidant effects in older adults with T2DM.

## 1. Introduction

Type 2 diabetes mellitus (T2DM) is a metabolic disorder that frequently occurs in older adults. Its pathophysiology has been linked to processes such as oxidative stress (OS) and alterations in markers associated with the hallmarks of aging, including chronic inflammation, telomere shortening, mitochondrial dysfunction, and genomic instability. Its high frequency and the debilitating nature of its complications pose challenges for healthcare systems worldwide [1,2,3,4,5,6,7,8,9].

Hence, there is a need to explore new therapeutic targets, with a focus on the pathophysiological mechanisms underlying T2DM, such as the activation of the family of enzymes structurally related to silent mating-type regulatory protein 2 (Sir2), better known as sirtuins (SIRTs) or longevity proteins. These enzymes are involved in DNA damage repair, stabilization of telomere shortening, regulation of the inflammatory process and glycemic metabolism, control of mitochondrial function, and protection against OS.

Among the nutritional interventions that improve glycemic response and have direct effects on sirtuins are those that focus on the use of polyphenols, bioactive compounds abundant in various fruits, such as berries, blueberries, raspberries, and grapefruit; and, as recently demonstrated in *Sechium edule* var. *nigrum spinosum* (which contains flavonoids with antioxidant activity and direct effects on the activation of certain sirtuins such as myricetin, naringenin, and quercetin), whose presence has been evidenced by our research group [10,11,12].

Previous studies have shown that consuming *Sechium edule* powder capsules has hypoglycemic, anti-inflammatory, hypotensive, and antioxidant effects in older adults with metabolic syndrome (MetS), with impacts at the molecular level through modulating the expression of mRNA of genes encoding enzymes with antioxidant functions and nuclear factor erythroid 2-related factor 2 (Nrf2) [13,14,15,16,17,18].

Sirtuins have been shown to deacetylate Nrf2, thereby promoting its activation and, consequently, the activation of antioxidant protection mechanisms, thereby reducing reactive oxygen species (ROS) levels [19]. Therefore, the objective of this study was to evaluate whether *Sechium edule* supplementation activates mRNA expression of genes encoding sirtuins in older adults with T2DM.

## 2. Results

Table 1 presents the anthropometric and clinical parameters, along with participants’ ages. No statistically significant differences were found in these measurements.

Glucose, cholesterol, triglyceride, HDL, uric acid, urea, albumin, and %HbA1c levels did not show statistically significant changes in either group at 3 or 6 months post-treatment (Table 2).

Regarding total oxidative capacity (Table 3), a statistically significant decrease was observed at six months post-treatment in the EG group (6.4 ± 2.9 (baseline) vs. 3.2 ± 2.1 (post)), which also showed a decrease in OSI (6.6 ± 3.3 (baseline) vs. 2.0 ± 1.4 (post)). Conversely, total antioxidant capacity increased (0.9 ± 0.3 (baseline) vs. 1.3 ± 0.2 (post)) in the same group.

**Figure 1:**
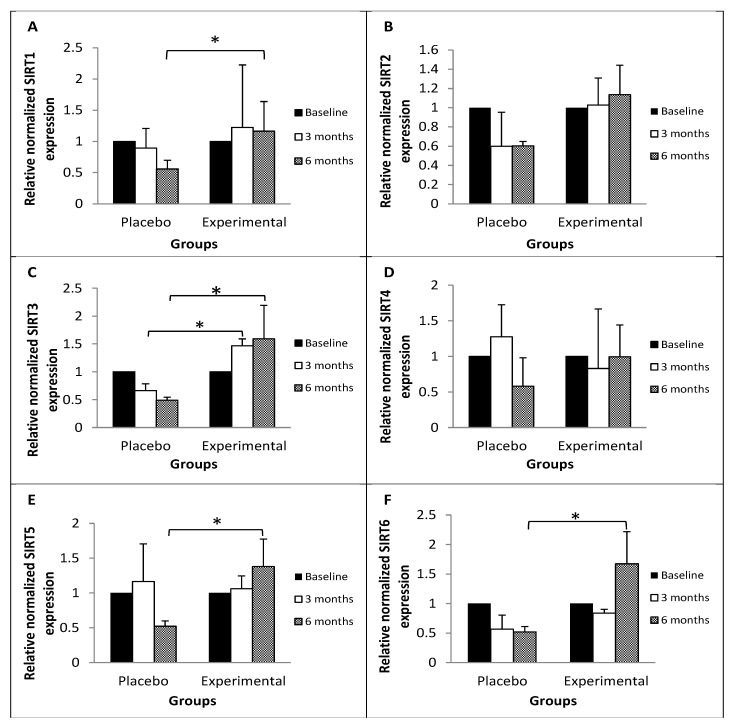
Relative mRNA expression of genes encoding proteins that coordinate the response to different types of metabolic or energy stress among the study subjects. Data are expressed as the mean ± standard deviation. Repeated measures ANOVA, significance level: 95%, *p* < 0.05. (**A**) SIRT1: sirtuin 1; (**B**) SIRT2: sirtuin 2; (**C**) SIRT3: sirtuin 3; (**D**) SIRT4: sirtuin 4; (**E**) SIRT5: sirtuin 5; (**F**) SIRT6: sirtuin 6. Relative mRNA expression levels were determined after normalization against β-actin. * Statistical significance between PG vs. EG at three- and six-month post-treatment intervals.

Figure 1 shows the relative expression of mRNA from genes encoding proteins that regulate responses to metabolic or energy stress. SIRT1 (Figure 1A), SIRT3 (Figure 1C), SIRT5 (Figure 1E), and SIRT6 (Figure 1F) showed statistically higher levels at 6 months post-treatment in the EG group compared to the PG group (52%, 69%, 62%, and 69% of EG levels, respectively).

## 3. Discussion

Type 2 diabetes mellitus (T2DM) is a metabolic disorder characterized by chronic hyperglycemia secondary to defects in insulin secretion and/or action. Individuals with T2DM have a greater predisposition to developing pathologies such as cerebrovascular and cardiac diseases, as well as infections, among others [20,21]. It is estimated that by 2050, approximately 1.31 billion people will suffer from T2DM [22]; therefore, this pathology is and will continue to be a serious public health problem worldwide. Hence, there is a need to prevent its complications.

*Sechium edule* is a fruit that has been attributed to have hypotensive, anti-inflammatory, hypoglycemic, antioxidant, and even geroprotective properties when administered to older adults with metabolic syndrome (MetS) [17,21,23,24,25]. Therefore, it is possible that *Sechium edule* could be used as an adjunct in the treatment of T2DM in older adults [26,27].

However, this investigation found no significant effect on clinical and biochemical markers in patients with T2DM. These findings can be explained by considering the specific differences between our population and those studied previously. It is important to note that the previous studies, although conducted in older adults, were performed in patients with MetS, who may present with hyperglycemia or glucose intolerance, but not with diabetes. Therefore, given the pathophysiology of T2DM, it is reasonable to assume that the effects of *Sechium edule* were insufficient to induce significant changes in this population. This proposal can be supported by the findings of a systematic review and meta-analysis showing that the hypoglycemic effect of *Sechium edule* is modest—an approximately 1% reduction in HbA1c levels [28], which is around 20 mg/dL, which may not be sufficient to generate a statistically significant change in patients with type 2 diabetes. However, studies suggest that any reduction in HbA1c decreases the risk of micro- and macrovascular complications and even death [29]. In this regard, we assume that, given the pathophysiology of T2DM, its natural history, and the development of metabolic memory, achieving significant changes in the identified markers is more complex. This explains why only some non-significant trends were observed in SBP, DBP, and HbA1c% levels.

On the other hand, it is interesting to observe the effect of *Sechium edule* on sirtuins and OS markers. Sirtuins are enzymes that belong to a family of seven members (SIRT1-SIRT7), with deacetylase (SIRT1-SIRT3, SIRT5-SIRT7) or ADP-ribosyltransferase (SIRT4 and SIRT6) activity, and are dependent on nicotinamide adenine dinucleotide (NAD+) and respond to various stressors, such as genotoxic and oxidative stressors. Accumulating evidence indicates that certain nutraceuticals present in a wide variety of fruits enhance sirtuin activity, leading to beneficial clinical outcomes in the treatment of cardiovascular diseases, arthritis, osteoporosis, dementia, and T2DM [30,31,32,33].

SIRT1 is a histone deacetylase that acts as a nutrient sensor. Its expression increases with caloric restriction and decreases with overfeeding. A decrease in SIRT1 expression leads to the recruitment or infiltration of macrophages into adipose tissue, resulting in histone hyperacetylation and, consequently, ectopic inflammatory expression. Conversely, its overexpression prevents this [34]. In human monocytes from patients with MetS, decreased SIRT1 expression levels have been associated with insulin resistance and atherosclerosis. Therefore, both glucotoxicity and lipotoxicity affect its expression [35]. In contrast, caloric restriction increases SIRT1 expression, which is associated with reduced inflammation and histological renal lesions in diabetic models. Consequently, it has been proposed as a promising therapy for preventing diabetic nephropathy with an anti-aging focus [36,37].

Along the same lines, it has been observed that certain nutraceuticals in *Sechium edule* enhance SIRT1 expression, thereby significantly improving mitochondrial function by mitigating OS and inflammation. Myricetin administration has been reported to promote mitochondrial biogenesis via SIRT1-mediated deacetylation of Peroxisome Proliferator-Activated Receptor Gamma Coactivator 1-alpha (PGC-1α) in experimental models [38].

It has also been observed that naringenin mitigates OS and inflammation and regulates mitochondrial function in human granulosa cells (KGN cells) by promoting SIRT1 expression [39]. Likewise, it has been reported that six months of resveratrol consumption increased SIRT1 levels by 50% in individuals with T2DM [30]. In the present study, we observed a 52% increase in SIRT1 mRNA expression in the EG group compared with the PG group, likely associated with the observed effects on oxidative markers [40].

It has been noted that SIRT1 activation increases the post-translational activity of Nrf2, a transcription factor that regulates antioxidant protection mechanisms [41]. Furthermore, *Sechium edule* has been shown to increase the mRNA levels of the genes encoding Nrf2 and antioxidant enzymes, such as superoxide dismutase (SOD) and catalase (CAT), in patients with MetS [18]. This agrees with the results of the present study, where we found a 44% increase in TAS in the EG group at six months post-treatment. This could suggest that consumption of Sechium edule improves antioxidant capacity by increasing SIRT1 expression.

On the other hand, our results showed that *Sechium edule* consumption had no effect on SIRT2 and SIRT4 expression, probably because these enzymes maintain a delicate balance to maintain cellular homeostasis, so both extremes (overexpression or downregulation) can be dangerous. SIRT2 is inhibited in people with T2DM. This effect restores pancreatic β-cells’ ability to enter the cell cycle and counteracts the decrease in the number of these cells [42]. For its part, SIRT4 overexpression leads to dyslipidemia, lipogenesis, and inhibition of insulin secretion [43]. At the same time, its absence increases insulin levels, leading to accelerated development of insulin resistance and OS in vivo models [44]. It can be seen that the effects of SIRT4 can vary, suggesting that its regulation is a controlled process in which the components of *Sechium edule* failed to exert any effect.

SIRT3, similar to SIRT1, regulates mitochondrial acetylation levels, protecting mitochondria from a wide range of damage, including oxidative injury [45,46]. Therefore, it is reasonable to assume that both sirtuins participate in similar protective mechanisms. It has been proposed that naringenin promotes mitochondrial biogenesis by reducing oxidative damage, thereby attenuating ischemia–reperfusion injury and cardiac damage via AMPK/SIRT3 signaling [47]. In cardiomyocytes from diabetic models, SIRT3 overexpression has been observed to attenuate hypertrophy and fibrosis and reduce ROS formation [48].

In the present study, we observed a 44% increase in TAS in the EG group, along with decreases in TOS and OSI (50% and 70%, respectively). This could indicate that *Sechium edule* supplementation promotes a balance between TOS/TAS levels, suggesting the maintenance of cellular homeostasis in these individuals and potentially leading to effective ROS clearance [49,50].

SIRT5, like SIRT3, promotes antioxidant defense mechanisms; its inactivation impairs NADPH production and reduces glutathione (GSH) synthesis. Therefore, the 62% increase in its expression in the EG group may be associated with the antioxidant effect observed in this group.

This behavior could be attributable to the presence of bioactive compounds in *Sechium edule*. One of them is quercetin, a flavonoid that promotes the desuccinylation of isocitrate dehydrogenase 2 (IDH2), a major source of NADPH, thereby supporting mitochondrial homeostasis, protecting against inflammation, and reducing oxidative damage [51]. In this regard, it is worth noting that supplementation with *Sechium edule* has been reported to reduce oxidative damage to lipids, proteins, and DNA [17].

Similarly, quercetin has been shown to activate not only SIRT5 but also SIRT6, thereby protecting against age-related metabolic diseases and regulating chromatin homeostasis for telomere maintenance [52,53]. Its decrease leads to telomere dysfunction, premature cellular senescence, and chromosome end fusions [54]. Our research group has shown that *consumption of Sechium edule* prevents telomere shortening [55]. Furthermore, like SIRT1, SIRT6 is essential for Nrf2 transcriptional activation under OS conditions. This reinforces the findings of the present study, which show an increase in TAS and decreases in TOS and OSI in the EG [56]. Therefore, the 69% increase in SIRT6 gene expression at six months post-treatment in the EG may also be related to greater antioxidant protection [57,58].

On the other hand, it is well established that NAD+ levels decrease during the aging process, leading to impaired nuclear and mitochondrial function [59]. Therefore, based on our results, we suggest that the antioxidant effect of *Sechium edule* supplementation may be associated with the restoration of NAD^+^ levels, as evidenced by the evident increase in sirtuin transcriptional levels. This points to an increase in deacetylation [60,61], favoring the maintenance of redox homeostasis, which is mediated by Nrf2. Similarly, sirtuins can directly deacetylate Nrf2 at lysine residues, promoting its nuclear translocation, its ability to bind the antioxidant response element (ARE) in DNA, and/or inhibiting its ubiquitination via Keap1 [62]. Furthermore, it has been noted that the flavonoids present in *Sechium edule* mimic the effects of caloric restriction (CRM) by modulating metabolic pathways, including AMPK (which detects cellular energy levels and increases NAD levels) [63]. Hence, a synergistic interaction could exist between the bioactive components of *Sechium edule*, involving the SIRT-AMPK-Nrf2 axis, which simultaneously integrates energy sensing and defenses against oxidative stress. This coincides with the observed results: increased expression of SIRT1, 3, 5, and 6 mRNA and increased total antioxidant/oxidative capacity.

One of the main limitations of this study is the very small, unrepresentative sample size and the variability in the data (very large standard deviations). Therefore, the reported results should be interpreted with caution, as they lack sufficient statistical power to generalize. For this reason, further clinical studies with representative samples and different clinical conditions are needed to confirm our findings.

## 4. Materials and Methods

### 4.1. Experimental Design

This study was approved by the Research and Biosafety Bioethics Committee of the Faculty of Higher Studies Zaragoza, UNAM (FESZ/DEPI/CE/023/22/; 21 October 2022), under trial registration number ISRCTN 43215432.

All procedures were conducted in accordance with the ethical principles of the Declaration of Helsinki of the World Medical Association. Informed consent was obtained from every participant. The fruits of *Sechium edule*, varietal group *nigrum spinosum*, were donated by the Interdisciplinary Research Group on *Sechium edule* A.C. (GISeM) of the RED-Chayote of the Agricultural Genetic Resources Subcommittee of the National Seed Inspection and Certification Service (NSICS), which focuses on the conservation, improvement, characterization, and enrichment of the genus *Sechium* in Mexico in the Municipality of Huatusco in the State of Veracruz; this is where the fruits used to make the capsules used in the study were harvested [64].

The fruit characterization was carried out according to the guidelines proposed by the International Union for the Protection of New Varieties of Plants (UPOV) and was validated, including morphological, phenotypic, and chromosomal characterization, by GISeM [16,64].

The biological material was collected at horticultural maturity (when the fruit is suitable for consumption), selected, washed, disinfected, and sectioned into slices, which were then dried at 40 °C and pulverized (epidermis, seeds, and spines). Previously, our research group identified the secondary metabolites present in each chayote capsule, which were determined using HPLC. These metabolites, in ascending order of concentration, were as follows: 0.71 μg of cucurbitacin I, 6.11 μg of cucurbitacin D, 89.9 μg of cucurbitacin B and 154.8 μg of cucurbitacin E; flavonoids: 0.014 μg of apigenin, 1.3 μg of quercetin, 2.38 μg of myricetin, 14.2 μg of phlorizin, 45.5 μg of rutin and 48.8 μg of naringenin; and phenolic acids: 0.11 μg of p-hydroxybenzoic, 1.4 μg of chlorogenic, 1.7 μg of p-coumaric, 3.3 μg of protocatechuic, 7.0 μg of ferulic, 8.7 μg of syringic, 9.3 μg of caffeic and 38.8 μg of gallic [17] (Supplementary Materials S1).

### 4.2. Intervention

The capsule formulation was designed in the pharmaceutical development laboratory of FES Zaragoza. The placebo was prepared using pharmaceutical-grade lactose monohydrate and talc (United States Pharmacopeia, USP) (Sigma, St. Louis, MO, USA). The optimal particle size of the *Sechium* powder was determined, and rheological studies were conducted to ensure capsule filling, weight, homogeneity, and stability. In accordance with the design, the treatments were manufactured and packaged by a pharmaceutical company specializing in nutraceutical products. The intervention consisted of consuming three capsules (placebo or active) per day (500 mg of powdered *Sechium edule*, one before each meal) for six months. For the selection of the *Sechium edule* dose, extrapolation between species (mouse and human) was performed using allometric scaling [65]. We also relied on data previously published by our research group, which used CD-1 strain mice as an experimental model and administered different doses of *Sechium edule* (8–5000 mg/kg) intraperitoneally; the LD_50_ was found to be greater than 5000 mg/kg. In this study, doses starting at 800 mg/kg did not cause significant changes in blood chemistry or toxicity in lymphoid organs, including the spleen and thymus, as well as the liver and kidneys. Furthermore, it significantly reduced glucose levels [66]. Subsequently, the dose was adjusted to a higher, intermediate level between 800 and 1600 mg/kg, that is, 1200 mg/kg. This is because intraperitoneal administration results in greater bioavailability compared to oral administration [67]. This dose was then extrapolated to humans, corresponding to 512.64 mg. Therefore, the dosage was adjusted to 500 mg per capsule. Subsequently, an exploratory study was conducted in which 12 older adults with MetS were administered three capsules of *Sechium edule* daily for six weeks, which demonstrated hepatoprotective, nephroprotective, and antioxidant effects after consumption of the fruit [28].

**Figure 2:**
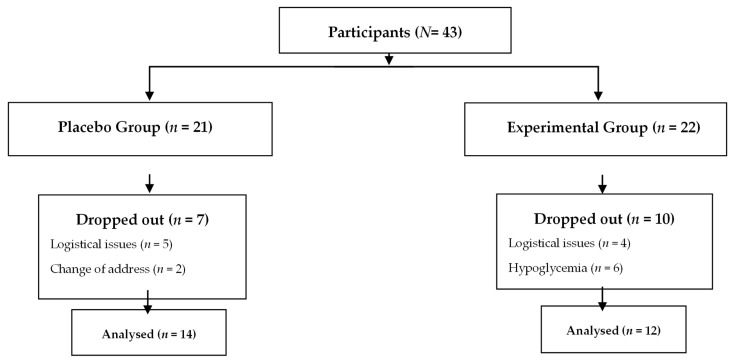
Flowchart of the participant monitoring process.

A quasi-experimental study was conducted in a convenience sample of 43 older adults, with a mean age of 66 ± 3 years. The participants were assigned to the (i) placebo group (PG; *n* = 21) or (ii) experimental group (EG; *n* = 22). Only some subjects agreed to donate venous blood for gene expression measurements during the study (PG, *n* = 14; EG, *n* = 12) (Figure 2). In the PG, seven patients dropped out of the study: five due to logistical issues (inability to attend measurements) and two due to a change in address. In the EG, ten people dropped out of the study: four due to logistical issues (inability to attend measurements) and six due to hypoglycemia. In both groups, all measurements were taken at baseline (before treatment) and at 3 and 6 months (post-treatment).

### 4.3. Anthropometric and Blood Pressure Measurements

Body weight (kg) and waist circumference (cm) were recorded. Body weight was determined using a calibrated medical scale (SECA, Hamburg, Germany), while waist circumference was measured at the level of the umbilicus with a medical measuring tape (SECA, Hamburg, Germany). These measurements were performed by trained nursing staff [68]. Systolic (SBP) and diastolic (DBP) blood pressure were measured using a calibrated mercury sphygmomanometer. Patients were asked to rest for at least five minutes before the measurement, seated in a chair with a backrest, with their back straight, legs uncrossed, and feet flat on the floor. Finally, the Osler technique was used to identify pseudohypertension [69].

### 4.4. Biochemical Analysis

For blood sampling, participants were asked to fast for at least 8 h. Samples were obtained by venipuncture and collected in vacuum tubes without anticoagulant. For clinical chemistry determinations (glucose, cholesterol, triglycerides, high-density lipoproteins (HDL-c), uric acid, urea, and albumin), colorimetric techniques were used with a Selectra Junior automated clinical chemistry analyzer (Vital Scientific, Dieren, The Netherlands). An immunoturbidimetric assay with the same clinical chemistry analyzer determined the percentage of glycated hemoglobin. Total antioxidants and oxidant status (TOS/TAS) were determined from heparinized plasma, and, finally, samples were collected in tubes containing the anticoagulant EDTA for lymphocyte isolation.

### 4.5. Total Oxidation Status (TOS)

The TOS was determined using a commercial kit (Rel Assay Diagnostics, Gaziantep, Turkey). In this kit, the oxidants present in the sample can oxidize the ferrous ion-chelating complex to ferric ions. In an acidic medium, this ion forms a colored complex with the chromogen, which can be measured spectrophotometrically. Therefore, the color intensity is directly associated with the amount of oxidants present in the sample. This test uses hydrogen peroxide (H_2_O_2_) as a calibrator.

### 4.6. Total Antioxidant Status (TAS)

The TAS was quantified using a kit (Randox Laboratories Ltd., Antrim, UK) that uses metmyoglobin and H_2_O_2_, along with 2,2-azino-bis (3-ethylbenzthiazoline-6-sulfonic acid) (ABTS), to produce a blue-green stain from the ABTS+ cationic radical. The ABTS+ cation is relatively stable and was measured at 600 nm. Color intensity is inversely proportional to the amount of antioxidants present in the sample.

### 4.7. Oxidative Stress Index (OSI)

The OSI was determined as the ratio of TOS to TAS (TOS/TAS) [70].

### 4.8. Lymphocyte Isolation and RNA Extraction

Lymphocyte separation was performed using 5 mL of venous blood diluted in equal parts sterile phosphate-buffered saline (PBS) (Sigma, St. Louis, MO, USA) and 2% fetal bovine serum (FBS) (ThermoFisher Scientific, Waltham, MA, USA). Four milliliters of Ficoll-Paque (Gibco ThermoFisher Scientific, Waltham, MA, USA) was added. The mixture was centrifuged at 200× *g*, and the opaque interface was transferred to a sterile tube. RNA extraction from 2 × 10^6^ lymphocytes was performed using the RNeasy Mini isolation kit (Qiagen, Hilden, Düsseldorf, Germany) according to the manufacturer’s recommendations, and the samples were stored at 70 °C until use. RNA was quantified and its integrity was determined by loading 5 µg on a 1% agarose gel with ethidium bromide and Tris-acetate-EDTA buffer, and visualized using Kodak Molecular Imaging software (v.4.5.1). Simultaneously, its purity was calculated using the A260/A280 ratio. All RNA samples used were considered intact and of optimal purity.

### 4.9. Gene Expression Analysis

All reactions were performed using 10 ng of RNA and forward and reverse primers at a final concentration of 100 nM (IDT, Coralville, IA, USA) (Table 4). The primers were generated using a primer design tool (NCBI Primer-BLAST tool from NIH) software (v.2.5.0) [71].

**Figure 3:**
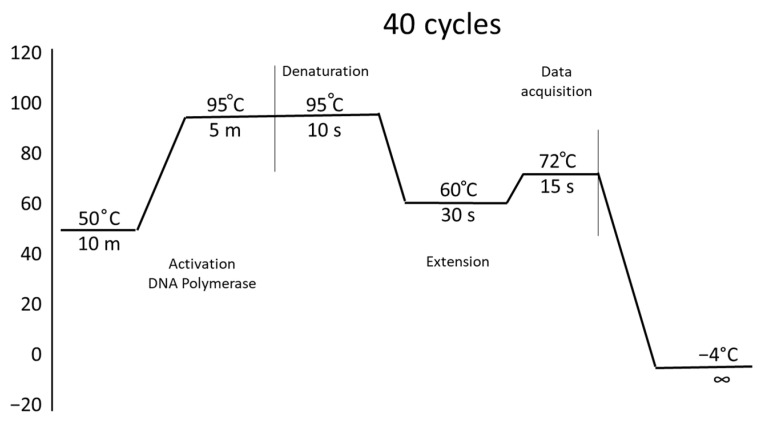
PCR stages. Temperatures, times, and cycles.

The QuantiFast SYBR Green RT-PCR kit (one-step RT-PCR) (Qiagen, Hilden, Düsseldorf, Germany) was also used for gene expression analysis, which allows for simultaneous execution of reverse transcription and PCR reactions. The reaction conditions are shown in Figure 3. The mean crossing threshold (Ct) of each gene was normalized to the mean Ct of the housekeeping gene β-actin. 

It should be noted that the gene selected for normalization by qPCR was based on a study, which compared different reference genes to evaluate transcription levels. In this study, they found that the β-actin and tyrosine 3-monooxygenase/tryptophan 5-monooxygenase activation protein zeta (YWHAZ) genes are the most stable genes in peripheral blood mononuclear cells of patients with type 2 diabetes mellitus (T2DM), which led us to select β-actin as the reference and control gene [72].

### 4.10. Statistical Analysis

The results are presented as the mean ± standard deviation and were analyzed using repeated measures ANOVA. Associations between sirtuin gene expression and the parameters analyzed were determined using Pearson’s correlation coefficient in statistical SPSS (IBM, Armonk, NY, USA) software (v.25). Results were considered statistically significant when *p* < 0.05. All determinations were performed in duplicate.

## 5. Conclusions

Our findings suggest that consumption of *Sechium edule* for six months increases the transcriptional expression of SIRT1, 3, 5, and 6; likewise, it improves the antioxidant response capacity, as evidenced by the reduction in OSI. Therefore, supplementation with this fruit could be useful as a complementary treatment to delay the development of complications, given its antioxidant effects and role as a metabolic regulator in older adults with type 2 diabetes. However, further studies with representative population samples in different clinical contexts and conditions are needed.

## Figures and Tables

**Table 1:** Anthropometric characteristics and blood pressure by study group.

Parameter	PG *n* = 14	EG *n* = 12	*p*-Value
Age (years)	67.1 ± 2.0	65.7 ± 4.8	
Weight (kg)			
Baseline	63.4 ± 2.5	69.1 ± 2.6	
Three months	63.7 ± 2.6	69.3 ± 2.7	0.16
Six months	64.4 ± 2.8	69.2 ± 2.9	0.25
BMI (kg/m^2^)			
Baseline	26.0 ± 3.8	30.0 ± 1.6	
Three months	26.3 ± 3.6	30.1 ± 2.2	0.06
Six months	26.2 ± 3.6	30.1 ± 2.7	0.06
Waist circumference (cm)			
Baseline	95.7 ± 2.1	103.4 ± 2.1	
Three months	95.6 ± 2.3	102.6 ± 2.3	0.06
Six months	95.8 ± 2.2	101.8 ± 2.2	0.07
SBP (mmHg)			
Baseline	127.7 ± 4.3	132.2 ± 4.3	
Three months	126.7 ± 6.3	126.5 ± 6.3	0.76
Six months	127.2 ± 6.3	129.4 ± 6.3	0.80
DBP (mmHg)			
Baseline	77.7 ± 2.5	82.7 ± 2.5	
Three months	79.7 ± 3.0	73.3 ± 3.0	0.15
Six months	80.0 ± 3.6	77.2 ± 3.6	0.59

Abbreviations: PG, placebo group; EG, experimental group; BMI, body mass index; SBP, systolic blood pressure; DBP, diastolic blood pressure. Data are expressed as means ± standard deviations. ANOVA of repeated measures test, significance level: 95%. Baseline vs. 3-month and baseline vs. 6-month inter-group *p*-values are shown.

**Table 2:** Biochemical parameters by study group.

Parameter	PG *n* = 14	EG *n* = 12	*p*-Value
Glucose (mg/dL)			
Baseline	182 ± 48	170 ± 42	
Three months	192 ± 75	164 ± 77	0.82
Six months	181 ± 38	170 ± 46	0.72
Cholesterol (mg/dL)			
Baseline	196 ± 49	183 ± 27	
Three months	200 ± 53	196 ± 18	0.86
Six months	161 ± 47	160 ± 45	0.99
Triglycerides (mg/dL)			
Baseline	164 ± 93	153 ± 86	
Three months	163 ± 54	151 ± 42	0.30
Six months	169 ± 67	139 ± 60	0.69
HDL-c (mg/dL)			
Baseline	47 ± 10	43 ± 10	
Three months	41 ± 8	41 ± 9	0.63
Six months	45 ± 10	44 ± 10	0.57
Uric acid (mg/dL)			
Baseline	4.84 ± 0.6	4.37 ± 0.6	
Three months	4.03 ± 1.2	4.63 ± 0.7	0.63
Six months	4.60 ± 1.5	4.87 ± 1.1	0.87
Urea (mg/dL)			
Baseline	32 ± 9.5	30 ± 7.8	
Three months	34 ± 9.0	30 ± 4.2	0.17
Six months	31 ± 9.0	27 ± 4.3	0.28
Albumin (g/dL)			
Baseline	3.9 ± 0.19	3.67 ± 0.14	
Three months	4.0 ± 0.28	3.94 ± 0.15	0.21
Six months	4.1 ± 0.09	4.06 ± 0.21	0.91
HbA1c (%)			
Baseline	6.98 ± 0.80	8.72 ± 1.47	
Three months	7.02 ± 0.83	9.25 ± 1.30	0.45
Six months	6.86 ± 0.76	7.46 ± 1.93	0.97

Abbreviations: PG, placebo group; EG, experimental group; HDL-c; high-density lipoprotein cholesterol; HbA1c, glycosylated hemoglobin. Data are expressed as means ± standard deviations. ANOVA of repeated measures test, significance level: 95%. Baseline vs. 3-month and baseline vs. 6-month inter-group *p*-values are shown.

**Table 3:** Oxidizing/antioxidant capacity and oxidative stress index by study group.

Parameter	PG *n* = 14	EG *n* = 12	*p*-Value
TOS (µmol H_2_O_2_ Equiv./L)			
Baseline	5.3 ± 2.3	6.4 ± 2.9	
Three months	4.9 ± 2.9	5.9 ± 3.0	0.63
Six months	4.7 ± 3.8	3.2 ± 2.1 *	0.03
TAS (mmol/L)			
Baseline	1.0 ± 0.3	0.9 ± 0.3	
Three months	1.1 ± 0.1	1.0 ± 0.2	0.86
Six months	1.0 ± 0.11	1.3 ± 0.2 *	0.04
OSI			
Baseline	6.0 ± 3.4	6.6 ± 3.3	
Three months	5.4 ± 3.6	4.9 ± 2.1	0.07
Six months	5.1 ± 4.4	2.0 ± 1.4 *	0.01

Abbreviations: PG, placebo group; EG, experimental group; TOS, total oxidation status; TAS, total antioxidant capacity; OSI, oxidative stress index. * Data are expressed as means ± standard deviations. ANOVA of repeated measures test, significance level: 95%. Baseline vs. 3-month and baseline vs. 6-month inter-group *p*-values are shown.

**Table 4:** Details of primers used for real-time PCR assays.

Gene	Primer Name	Sequence
SIRT1	SIRT1-F SIRT1-R	GGGCTGCGGTTCCTACTG TTATCTGGCTGCTGCGGAAA
SIRT2	SIRT2-F SIRT2-R	CTCTCACCCTCTGGAGACCC ATGTCTGCTTCTCCACCAGC
SIRT3	SIRT3-F SIRT3-R	GGTAGTTGAACGGGTCGAGG TAATAATCGTCCCTGCCGCC
SIRT4	SIRT4-F SIRT4-R	CAATCAGACGGTCCCACTGT ATCCAACGGCCTTTTGCTGA
SIRT5	SIRT5-F SIRT5-R	ACGTCGTGTGGTTTGGAGAA GGAAGTGCCCACCACTAGAC
SIRT6	SIRT6-F SIRT6-R	GCAGTCTTCCAGTGTGGTGT TCCTCCATGGTCCAGACTCC
β-ACTIN	ACTIN-F ACTIN-R	GAGCACAGAGCCTCGCC CGCGGCGATATCATCATCCA

SIRT1: (silent mating type information regulation 2 homolog 1 (*S. cerevisiae*), human; SIRT2: silent mating type information regulation 2 homolog 2 (*S. cerevisiae*) protein, human; SIRT3: silent mating type information regulation 2 homolog 3 (*S. cerevisiae*) protein, human; SIRT4: silent mating type information regulation 2 homolog 4, human; SIRT5: silent mating type information regulation 2 homolog 5, human; SIRT6: silent mating type information regulation 2 homolog 6 protein, human.

## Data Availability

The data that support the findings of this study are available from the corresponding author [V.M.M.-N.] upon reasonable request.

## Supplementary Materials

The following supporting information can be downloaded at: https://www.mdpi.com/article/10.3390/molecules31071182/s1. Supplementary Materials S1. Identification of secondary metabolites by High-Performance Liquid Chromatography (HPLC). References [66,73] are cited in the supplementary materials.

## References

[1] Mendoza-Núñez V.M., Martínez-Maldonado M.L., Vivaldo-Martínez M. (2016). What is the onset age of human aging and old age?. Int. J. Gerontol..

[2] WHO Ageing and Health. https://www.who.int/news-room/fact-sheets/detail/ageing-and-health.

[3] López-Otín C., Blasco M.A., Partridge L., Serrano M., Kroemer G. (2023). Hallmarks of aging: An expanding universe. Cell.

[4] López-Otín C., Kroemer G. (2025). Hallmarks of aging: Integrating molecular and social determinants. Geromedicine.

[5] WHO Diabetes. https://www.who.int/news-room/fact-sheets/detail/diabetes.

[6] Yaribeygi H., Sathyapalan T., Atkin S.L., Sahebkar A. (2020). Molecular mechanisms linking oxidative stress and Diabetes Mellitus. Oxid. Med. Cell. Longev..

[7] Caturano A., D’Angelo M., Mormone A., Russo V., Mollica M.P., Salvatore T., Galiero R., Rinaldi L., Vetrano E., Marfella R. (2023). Oxidative stress in Type 2 Diabetes: Impacts from pathogenesis to lifestyle modifications. Curr. Issues Mol. Biol..

[8] Yaribeygi H., Mohammadi M.T., Sahebkar A. (2018). Crocin potentiates antioxidant defense system and improves oxidative damage in liver tissue in diabetic rats. Biomed. Pharmacother..

[9] Bellary S., Kyrou I., Brown J.E. (2021). Type 2 diabetes mellitus in older adults: Clinical considerations and management. Nat. Rev. Endocrinol..

[10] Kitada M., Ogura Y., Monno I., Koya D. (2019). Sirtuins and Type 2 Diabetes: Role in inflammation, oxidative stress, and mitochondrial function. Front. Endocrinol..

[11] Lingappa N., Mayrovitz H.N. (2022). Role of sirtuins in Diabetes and age-related processes. Cureus.

[12] NLM Sirtuins. https://www.ncbi.nlm.nih.gov/mesh/68037761.

[13] Abdelhaleem I.A., Brakat A.M., Adayel H.M., Asla M.M., Rizk M.A., Aboalfetoh A.Y. (2022). The effects of resveratrol on glycemic control and cardiometabolic parameters in patients with T2DM: A systematic review and meta-analysis. Med. Clin..

[14] Cao M.M., Lu X., Liu G.D., Su Y., Li Y.B., Zhou J. (2018). Resveratrol attenuates type 2 diabetes mellitus by mediating mitochondrial biogenesis and lipid metabolism via Sirtuin type 1. Exp. Ther. Med..

[15] Vieira E.F., Souza S., Moreira M.M., Cruz R., Silva A.B.D., Casal S., Delerue-Matos C. (2022). Valorization of phenolic and carotenoid compounds of *Sechium edule* (Jacq. Swartz) leaves: Comparison between conventional, ultrasound- and microwave-assisted extraction approaches. Molecules.

[16] Avendaño A.C.H., Cadena I.J., Arévalo G.M.L., Campos R.E., Cisneros S.V.M., Aguirre M.J.F. (2010). Las Variedades del Chayote Mexicano, Recurso Ancestral con Potencial de Comercialización.

[17] Arista-Ugalde T.L., Santiago-Osorio E., Monroy-García A., Rosado-Pérez J., Aguiñiga-Sánchez I., Cadena-Iñiguez J., Gavia-García G., Mendoza-Núñez V.M. (2022). Antioxidant and anti-inflammatory effect of the consumption of powdered concentrate of *Sechium edule* var. nigrum spinosum in Mexican older adults with metabolic syndrome. Antioxidants.

[18] Gavia-García G., Hernández-Álvarez D., Arista-Ugalde T.L., Aguiñiga-Sánchez I., Santiago-Osorio E., Mendoza-Núñez V.M., Rosado-Pérez J. (2023). The supplementation of *Sechium edule* var. nigrum spinosum (chayote) promotes Nrf2-mediated antioxidant protection in older adults with metabolic syndrome. Nutrients.

[19] Patel S., Khan H., Majumdar A. (2022). Crosstalk between Sirtuins and Nrf2: SIRT1 activators as emerging treatment for diabetic neuropathy. Metab. Brain Dis..

[20] WHO Classification of Diabetes Mellitus. https://www.who.int/publications/i/item/classification-of-diabetes-mellitus.

[21] Galicia-Garcia U., Benito-Vicente A., Jebari S., Larrea-Sebal A., Siddiqi H., Uribe K.B., Ostolaza H., Martín C. (2020). Pathophysiology of Type 2 Diabetes Mellitus. Int. J. Mol. Sci..

[22] GBD 2021 Diabetes Collaborators (2023). Global, regional, and national burden of diabetes from 1990 to 2021, with projections of prevalence to 2050: A systematic analysis for the Global Burden of Disease Study 2021. Lancet.

[23] Lombardo-Earl G., Roman-Ramos R., Zamilpa A., Herrera-Ruiz M., Rosas-Salgado G., Tortoriello J., Jiménez-Ferre E. (2014). Extracts and fractions from edible roots of *Sechium edule* (Jacq.) Sw. with antihypertensive activity. Evid. Based Complement. Alternat. Med..

[24] Gordon E.A., Guppy L.J., Nelson M. (2000). The antihypertensive effects of the Jamaican Cho-Cho (*Sechium edule*). West Indian Med. J..

[25] Rosado-Pérez J., Aguiñiga-Sánchez I., Santiago-Osorio E., Mendoza-Núñez V.M. (2019). Effect of *Sechium edule* var. nigrum spinosum (Chayote) on oxidative stress and pro-inflammatory markers in older adults with metabolic syndrome: An exploratory study. Antioxidants.

[26] Agbabiaka T., Wider B., Watson L.K., Goodman C. (2017). Concurrent use of prescription drugs and herbal medicinal products in older adults: A systematic review. Drugs Aging.

[27] Gavia-García G., Rosado-Pérez J., Aguiñiga-Sánchez I., Santiago-Osorio E., Mendoza-Núñez V.M. (2020). Effect of *Sechium edule* var. nigrum spinosum (Chayote) on telomerase levels and antioxidant capacity in older adults with metabolic syndrome. Antioxidants.

[28] Arista-Ugalde T.L., Delgado-Arroyo S., Gavia-García G., Hernández-Álvarez D., Aguiñiga-Sánchez I., Santiago-Osorio E., Rosado-Pérez J., Mendoza-Núñez V.M. (2025). Hypoglycemic effects of *Sechium edule* (Chayote) in older adults: A systematic review and meta-analysis of clinical and preclinical trials. Foods.

[29] Stratton I.M., Adler A.I., Neil H.A.W., Matthews D.R., Manley S.E., Cull C.A., Hadden D., Turner R.C., Holman R.R. (2000). Association of glycaemia with macrovascular and microvascular complications of type 2 diabetes (UKPDS 35): Prospective observational study. BMJ.

[30] García-Martínez B.I., Ruiz-Ramos M., Pedraza-Chaverri J., Santiago-Osorio E., Mendoza-Núñez V.M. (2023). Effect of resveratrol on markers of oxidative stress and sirtuin 1 in elderly adults with type 2 diabetes. Int. J. Mol. Sci..

[31] Grabowska W., Sikora E., Bielak-Zmijewska A. (2017). Sirtuins, a promising target in slowing down the ageing process. Biogerontology.

[32] Morris B.J. (2013). Seven sirtuins for seven deadly diseases of ageing. Free Radic. Biol. Med..

[33] Grabowska W., Suszek M., Wnuk M., Lewinska A., Wasiak E., Sikora E., Bielak-Zmijewska A. (2016). Curcumin elevates sirtuin level but does not postpone in vitro senescence of human cells building the vasculature. Oncotarget.

[34] Gillum M.P., Kotas M.E., Erion D.M., Kursawe R., Chatterjee P., Nead K.T., Muise E.S., Hsiao J.J., Frederick D.W., Yonemitsu S. (2011). Sirt1 regulates adipose tissue inflammation. Diabetes.

[35] de Kreutzenberg S.V., Ceolotto G., Papparella I., Bortoluzzi A., Semplicini A., Dalla M.C., Cobelli C., Fadini G.P., Avogaro A. (2010). Downregulation of the longevity-associated protein sirtuin 1 in insulin resistance and metabolic syndrome: Potential biochemical mechanisms. Diabetes.

[36] Li Y., Miao Y., Feng Q., Zhu W., Chen Y., Kang Q., Wang Z., Lu F., Zhang Q. (2024). Mitochondrial dysfunction and onset of type 2 diabetes along with its complications: A multi-omics Mendelian randomization and colocalization study. Front. Endocrinol..

[37] Kitada M., Takeda A., Nagai T., Ito H., Kanasaki K., Koya D. (2011). Dietary restriction ameliorates diabetic nephropathy through anti-inflammatory effects and regulation of the autophagy via restoration of Sirt1 in diabetic Wistar fatty (fa/fa) rats: A model of type 2 diabetes. Exp. Diabetes Res..

[38] Jung H.Y., Lee D., Ryu H.G., Choi B.H., Go Y., Lee N., Lee D., Son H.G. (2017). Myricetin improves endurance capacity and mitochondrial density by activating SIRT1 and PGC-1α. Sci. Rep..

[39] Yuan B., Mao J., Wang J., Luo S., Luo B. (2024). Naringenin mitigates cadmium-induced cell death, oxidative stress, mitochondrial dysfunction, and inflammation in KGN cells by regulating the expression of sirtuin-1. Drug Chem. Toxicol..

[40] Rodgers J.T., Lerin C., Haas W., Gygi S.P., Spiegelman B.M., Puigserver P. (2005). Nutrient control of glucose homeostasis through a complex of PGC-1 alpha and SIRT1. Nature.

[41] Gureev A.P., Krutskikh E.P. (2025). Regulation of the NRF2 transcription factor activity by SIRT1-induced deacetylation: A possible SIRT1–NRF2 feedback loop. Russ. J. Genet..

[42] Katz L.S., Scott D.K., Stewart A.F. (2025). SIRT2 puts the brakes on human β cell proliferation: Therapeutic opportunities and next challenges. J. Clin. Investig..

[43] Choubey S.K., Prabhu D., Nachiappan M., Biswal J., Jeyakanthan J. (2017). Molecular modeling, dynamics studies and density functional theory approaches to identify potential inhibitors of SIRT4 protein from Homo sapiens: A novel target for the treatment of type 2 diabetes. J. Biomol. Struct. Dyn..

[44] Huynh F.K., Hu X., Lin Z., Johnson J.D., Hirschey M.D. (2018). Loss of sirtuin 4 leads to elevated glucose- and leucine-stimulated insulin levels and accelerated age-induced insulin resistance in multiple murine genetic backgrounds. J. Inherit. Metab. Dis..

[45] Zhang J., Xiang H., Liu J., Chen Y., He R.R., Liu B. (2020). Mitochondrial Sirtuin 3: New emerging biological function and therapeutic target. Theranostics.

[46] Bellizzi D., Rose G., Cavalcante P., Covello G., Dato S., De Rango F., Greco V., Maggiolini M., Feraco E., Mari V. (2005). A novel VNTR enhancer within the SIRT3 gene, a human homologue of SIR2, is associated with survival at oldest ages. Genomics.

[47] Yu L.M., Dong X., Xue X.D., Zhang J., Li Z., Wu H.J., Yang Z.L., Yang Y., Wang H.S. (2019). Naringenin improves mitochondrial function and reduces cardiac damage following ischemia-reperfusion injury: The role of the AMPK-SIRT3 signaling pathway. Food Funct..

[48] Li L., Zeng H., He X., Chen J.X. (2021). Sirtuin 3 alleviates diabetic cardiomyopathy by regulating TIGAR and cardiomyocyte metabolism. J. Am. Heart Assoc..

[49] Zhou L., Wang F., Sun R., Chen X., Zhang M., Xu Q., Wang Y., Wang S., Xiong Y., Guan K.L. (2016). SIRT5 promotes IDH2 desuccinylation and G6PD deglutarylation to enhance cellular antioxidant defense. EMBO Rep..

[50] Zhou B., Yang Y., Pang X., Shi J., Jiang T., Zheng X. (2023). Quercetin inhibits DNA damage responses to induce apoptosis via SIRT5/PI3K/AKT pathway in non-small cell lung cancer. Biomed. Pharmacother..

[51] Chang X., Zhang T., Wang J., Liu Y., Yan P., Meng Q., Yin Y., Wang S. (2021). SIRT5-related desuccinylation modification contributes to quercetin-induced protection against heart failure and high-glucose-prompted cardiomyocytes injured through regulation of mitochondrial quality surveillance. Oxid. Med. Cell. Longev..

[52] You W., Zheng W., Weiss S., Chua K., Steegborn C. (2019). Structural basis for the activation and inhibition of Sirtuin 6 by quercetin and its derivatives. Sci. Rep..

[53] Zhang H., Zhang J., Zhang H.X. (2024). Effect of quercetin on the protein-substrate interactions in SIRT6: Insight from MD simulations. J. Mol. Graph. Model..

[54] Michishita E., McCord R.A., Berber E., Kioi M., Padilla-Nash H., Damian M., Cheung P., Kusumoto R., Kawahara T.L., Barrett J.C. (2008). SIRT6 is a histone H3 lysine 9 deacetylase that modulates telomeric chromatin. Nature.

[55] Gavia-García G., Rosado-Pérez J., Arista-Ugalde T.L., Aguiñiga-Sánchez I., Santiago-Osorio E., Mendoza-Núñez V.M. (2023). The consumption of *Sechium edule* (chayote) has antioxidant effect and prevents telomere attrition in older adults with metabolic syndrome. Redox Rep..

[56] Rezazadeh S., Yang D., Tombline G., Simon M., Regan S.P., Seluanov A., Gorbunova V. (2019). SIRT6 promotes transcription of a subset of NRF2 targets by mono-ADP-ribosylating BAF170. Nucleic Acids Res..

[57] Kuang J., Zhang Y., Liu Q., Shen J., Pu S., Cheng S., Chen L., Li H., Wu T., Li R. (2017). Fat-specific Sirt6 ablation sensitizes mice to high-fat diet–induced obesity and insulin resistance by inhibiting lipolysis. Diabetes.

[58] Kuang J., Chen L., Tang Q., Zhang J., Li Y., He J. (2018). The role of Sirt6 in obesity and diabetes. Front. Physiol..

[59] Imai S., Guarente L. (2014). NAD+ and sirtuins in aging and disease. Trends Cell Biol..

[60] Vedantham S., Thiagarajan D., Ananthakrishnan R., Wang L., Rosario R., Zou Y.S., Goldberg I., Yan S.F., Schmidt A.M., Ramasamy R. (2014). Aldose reductase drives hyperacetylation of Egr-1 in hyperglycemia and consequent upregulation of proinflammatory and prothrombotic signals. Diabetes.

[61] Kosanam H., Thai K., Zhang Y., Advani A., Connelly K.A., Diamandis E.P., Gilbert R.E. (2014). Diabetes induces lysine acetylation of intermediary metabolism enzymes in the kidney. Diabetes.

[62] Perez-Lao E.J., Fagerli E., Ferrier F., Young J.I., Perez-Pinzon M.A. (2025). Regulatory dynamics of Nrf2 with sirtuins in the brain: Exploring cellular metabolism, synaptic plasticity, and defense mechanisms. J. Neurochem..

[63] Murillo-Cancho A.F., Nievas-Soriano B.J., Lozano-Paniagua D. (2025). Miméticos de la restricción energética: Un análisis comparativo de agentes naturales y sintéticos en la modulación de la autofagia. Ars. Pharm..

[64] Cadena-Iñiguez J., Ruiz-Posadas L.M., Soto-Hernández M., Aguirre-Medina J.F., Avendaño-Arrazate C.H., Arévalo-Galarza L. (2008). Infraspecific variation of *Sechium edule* (Jacq.) Sw. in the state of Veracruz, Mexico. Genet. Resour. Crop. Evol..

[65] Nair A.B., Jacob S. (2016). A simple practice guide for dose conversion between animals and human. J. Basic Clin. Pharm..

[66] Aguiñiga-Sánchez I., Cadena-Íñiguez J., Santiago-Osorio E., Gómez-García G., Mendoza-Núñez V.M., Rosado-Pérez J., Ruíz-Ramos M., Cisneros-Solano V.M., Ledesma-Martínez E., Delgado-Bordonave A.J. (2017). Chemical analyses and in vitro and in vivo toxicity of fruit methanol extract of *Sechium edule* var. *nigrum spinosum*. Pharm. Biol..

[67] Al Shoyaib A., Archie S.R., Karamyan V.T. (2019). Intraperitoneal route of drug administration: Should it be used in experimental animal studies?. Pharm. Res..

[68] Secretaría de Salud (2002). Toma de Medidas Clínicas y Antropométricas en el Adulto Mayor.

[69] Secretaría de Salud (1999). Norma Oficial Mexicana NOM-030-SSA-1999. Para la Prevención, Tratamiento y Control de la Hipertensión Arterial.

[70] Sánchez-Rodríguez M.A., Mendoza-Núñez V.M. (2019). Oxidative stress indexes for diagnosis of health or disease in humans. Oxid. Med. Cell. Longev..

[71] Primer-BLAST-NCBI-NIH (Primer-BLAST). https://www.ncbi.nlm.nih.gov/tools/primer-blast/.

[72] Hazarika A., Nongkhlaw B., Mukhopadhyay A. (2023). Identification of stable reference genes in peripheral blood mononuclear cells from type 2 diabetes mellitus patients. Sci. Rep..

[73] Arista-Ugalde T.L. (2023). Efecto del Consumo de Frutos de *Sechium edule* Sobre Marcadores de Estrés Oxidativo, Inflamación Crónica y Daño Oxidativo al ADN en Adultos Mayores Con Síndrome Metabólico. Ph.D. Thesis.

