# Supplementary material for: Sechium edule var. nigrum spinosum (Chayote) Increases the mRNA Expression of Genes Encoding Sirtuins in Older Adults with Type 2 Diabetes Mellitus
Source: Molecules. 2026 Apr 2;31(7):1182. doi: 10.3390/molecules31071182 (PMC13074737; doi:10.3390/molecules31071182)
Supplement: Supplementary file 1 [file molecules-31-01182-s001.zip › molecules-4188548-supplementary.pdf]

Supplementary Materials S1.
Identification of secondary metabolites by High-Performance Liquid Chromatography (HPLC)

For metabolite identification, a Sechium edule capsule (500 mg of dry powder) was used, to which 1 mL of HPLC-grade methanol (Sigma-Aldrich, USA) was added. The sample was homogenized for one minute and allowed to stand for 14 days. The supernatant was recovered by filtration through a 0.45 µm membrane (Milipore, Ireland). All the filtered supernatant was placed in an HPLC vial. This procedure was performed in duplicate. Cucurbitacins were analyzed on a Symmetry Shield C18 column (4.6 x 250 mm) (Waters, Spain) by isocratic analysis in water:methanol: acetonitrile (50:30:20) solvents; with a flow rate of 1 mL min^{-1}; temperature 25°C; injection volume 40 µL; Detection was performed at 235 nm and the analysis time was 60 minutes (shown in the table). Cucurbitacin E, I, B, and D (Sigma-Aldrich, USA) were used as reference standards [Aguiñiga-Sánchez, 2017].

Retention time and wavelength for Cucurbitacin standards

**Compound**	**Retention Time (min) λ 235 nm**
Cucurbitacin B	38.83
Cucurbitacin D	15.69
Cucurbitacin E	52.87
Cucurbitacin I	19.79

For the detection of both phenolic acids and flavonoids, an Agilent 1100 liquid chromatograph was used, equipped with a 1200 autosampler and a 1100 diode array detector. A Hewlett-Packard Hypersyl ODS column (125 × 40 mm) was used. The mobile phase consisted of: a) water, pH 2.5, with trifluoroacetic acid, and b) acetonitrile. The analysis was performed by gradient induction: 15–35% B for 0.1–min, 35% B for 20–25 min. The flow rate was 1 mL min^{-1}, the temperature was 30°C, and the injection volume was 15–20 µL. The detector was set to λ1 254 nm, λ2 280 nm, λ3 330 nm, and λ4 365 nm at different retention times, with an analysis time of 25 min.

Retention time and λ for phenolic acid standards

**Compound**	**Retention Time (min)**
λ 254 nm	
Ácido protocatecuico	2.09
Ácido p-hidroxibenzoico	2.56
Ácido vainíllico o caféico	3.39
Ácido b-resorcílico	4.26
Ácido 3,5-di-hidroxibenzoico	11.27
λ 280 nm	
Ácido gálico	1.55
Ácido siríngico	3.00

Retention time and λ for flavonoid standards

**Compound**	**Retention Time (min)**
λ 254 nm	
Rutin	2.09
Morina	2.56
Quercetin	3.39
λ 280 nm	
Catechin	2.43
Hesperidin	8.25
Floridtzina	9.06
Naringenin	15.8

Ácido p-cumárico	5.29
λ 330 nm	
Ácido clorogénico	3.70
Ácido sináptico	5.96
Ácido ferúlico	6.10
Ácido rosmárico	9.27

Floretin	16.2
λ 330 nm	
Apigenin	17.1
λ 365 nm	
Myricetin	9.1
Kaempferol	17.5
Isorhamnetin	18.2

The detector was programmed to obtain the absorption spectra of each component of the analyzed samples and compare them with the spectra of the standards. Based on the absorption maxima, calibration curves were generated for the four different programmed wavelengths (λ). The reference standards used were from Sigma-Aldrich (USA). The flavonoid standards were rutin, quercetin, phlorizidin, myricetin, naringenin, phloretin, and apigenin. Finally, the results determined the concentration of the compounds based on the injection volume and the micrograms detected. The terpene fraction was used as a reference concentration of 2 mg/mL, and the flavonoid fraction was used as a reference concentration of 40 mg/mL [Arista-Ugalde, 2023].

The following table shows the content of secondary metabolites present in the dried fruit of Sechium edule, varietal group nigrum spinosum, per chayote capsule. The analysis was performed in duplicate.

**Metabolite name**	**Concentration [μg]**	**Standard deviation**
**Flavonoids**		
Rutin	45.49	12.44
Quercetin	1.30	0.176
Apigenin	0.014	0.007
Myricetin	2.38	0.129
Phorizin	14.26	2.28
Naringenin	48.85	7.046
**Phenolic acids**		
Syringic acid	8.7	0.034
Protocatechuic acid	3.3	1.56
Caffeic acid	9.27	0.066
p-Hydroxybenzoic acid	0.115	0.019
Gallic acid	38.83	1.37
p-Coumaric acid	1.69	0.23
Chlorogenic acid	1.39	0.30
Ferulic acid	7.03	0.37
**Cucurbitacins**		
Cucurbitacin D	6.11	0.63
Cucurbitacin B	89.94	18.15
Cucurbitacin E	154.8	3.3
Cucurbitacin I	0.71	0.26

Aguiñiga-Sánchez, I.; Cadena-Íñiguez, J.; Santiago-Osorio, E.; Gómez-García, G.; Mendoza-Núñez, V.M.; Rosado-Pérez, J.; Ruíz-Ramos, M.; Cisneros-Solano, V.M.; Ledesma-Martínez, E.; Delgado-Bordonave, A.J.; Soto-Hernández, R.M. Chemical analyses and in vitro and in vivo toxicity of fruit methanol extract of Sechium edule var. nigrum spinosum. Pharm. Biol. 2017, 55, 1638-1645.

Arista-Ugalde, T.L. Efecto del consumo de frutos de *Sechium edule* sobre marcadores de estrés oxidativo, inflamación crónica y daño oxidativo al ADN en adultos mayores con síndrome metabólico [tesis doctoral]. México: Dirección General de Bibliotecas. Universidad Nacional Autónoma de México, 2023.
